# Introducing Entropy into Organizational Psychology: An Entropy-Based Proactive Control Model

**DOI:** 10.3390/bs14010054

**Published:** 2024-01-15

**Authors:** Haozhe Jia, Lei Wang

**Affiliations:** School of Psychological and Cognitive Sciences and Beijing Key Lab for Behavior and Mental Health, Peking University, Beijing 100871, China; jiahzh@stu.pku.edu.cn

**Keywords:** psychological entropy, learning orientation, goal orientation, change orientation, risk taking

## Abstract

This paper provides a systematic review of the transfer and quantification of the concept of entropy in multidisciplinary fields and delves into its future applications and research directions in organizational management psychology based on its core characteristics. We first comprehensively reviewed the conceptual evolution of entropy in disciplines such as physics, information theory, and psychology, revealing its complexity and diversity as an interdisciplinary concept. Subsequently, we analyzed the quantification methods of entropy in a multidisciplinary context and pointed out that their calculation methods have both specificity and commonality across different disciplines. Subsequently, the paper reviewed the research on how individuals cope with uncertainty in entropy increase, redefined psychological entropy from the perspective of organizational management psychology, and proposed an “entropy-based proactive control model” at the individual level. This model is built around the core connotation of entropy, covering four dimensions: learning orientation, goal orientation, change orientation, and risk taking. We believe that psychological entropy, as a meta structure of individuals, can simulate, explain, and predict the process of how individuals manage and control “entropy” in an organizational environment from a dynamic perspective. This understanding enables psychological entropy to integrate a series of positive psychological constructs (e.g., lean spirit), providing extensive predictive and explanatory power for various behaviors of individuals in organizations. This paper provides a new direction for the application of the concept of entropy in psychology, especially for theoretical development and practical application in the field of organizational management.

## 1. Introduction

In the VUCA (volatility, uncertainty, complexity, ambiguity) era, organizations face unprecedented changes and challenges [1]. In such an environment, effectively responding to and managing instability and uncertainty from the external environment, as well as the resulting complexity and ambiguity, is crucial for the sustainable development of organizations.

Entropy, as a concept describing the ambiguity, uncertainty, and degree of chaos in systems, may offer a novel perspective in understanding the internal and external complexities of organizations and assist in exploring potential pathways to maintain organizational dynamism and systemic stability [2]. At the micro level, humans, as organic life systems, inherently engage in an entropy-resisting process in their survival and development, characterized by an innate tendency toward entropy reduction [3]. At the macro level, organizations as a whole need to proactively face continuously changing external challenges through resource allocation and process optimization to maintain competitiveness and innovation capability in a dynamic environment. Whether at the individual or organizational level, to achieve high-quality survival and development, the effective management of entropy (possessing the laws/processes of entropy reduction) is essential. Therefore, this article attempts to guide individuals and organizations in maintaining and enhancing adaptability and innovative capacity in ambiguous, disordered, complex, and uncertain environments by understanding and applying the concept of entropy.

Clausius first introduced the concept of entropy within the field of thermodynamics in 1865 [4]. He posited that entropy reflects the degree of energy’s even distribution. As the scientific community’s understanding and focus on entropy deepened, the concept was introduced into the field of information theory. In this domain, entropy is an information-theoretic measure of uncertainty based on a set of known event probabilities that is used to measure the amount of information (complexity) or uncertainty [5,6]. High information entropy signifies greater uncertainty, implying that the probability of all events tends to be uniform. Conversely, when the probability of a subset of events becomes greater than that of other events, information entropy correspondingly decreases. For instance, it is challenging to predict the outcome of a dice roll, which possesses a high degree of uncertainty, and hence, the information entropy is increased. In contrast, the natural law of the sun rising in the east and setting in the west has a fixed and unique orientation, thereby resulting in lower information entropy [7,8]. The introduction of information entropy not only brought the concepts of disorder and uncertainty into the understanding of entropy for the first time but also marked the interdisciplinary expansion of the concept of thermodynamic entropy [9,10]. This development paved the way for the application of entropy in various disciplinary fields, especially in providing new perspectives and theoretical frameworks for understanding and addressing the complexity and uncertainty of the VUCA era.

Although the concept of entropy has been transferred in various forms across multiple fields, its interdisciplinary application still faces numerous challenges. Taking psychology (specifically, organizational management psychology) as an example, first, there remains an incomplete understanding of key concepts and their ambiguities and subtleties. This partial comprehension may lead to vague interpretations of a range of psychological phenomena, as psychological phenomena themselves often exhibit an inherent complexity that is difficult to clearly explain with external concepts [11]. This ambiguity leads to theoretical uncertainty and poses difficulties for empirical research [12]. Second, the operation of organizations reveals a complex duality between order and disorder: they both disintegrate and organize; they are simultaneously complementary and competitive; and they constrain and promote each other. Therefore, both entropy increase (systems tending toward disorder) and entropy reduction (tending toward order) are fundamental to the existence and survival of organizations [13]. However, existing entropy theoretical frameworks have not fully addressed the synergistic transformation between order and chaos. These frameworks often adopt a deterministic perspective, which simplistically categorizes disorder and order, as well as chaos and organization, as completely contrasting concepts. Third, the organizational decision-making process involves diverse individual behaviors and complex organizational dynamics, encompassing entropy reduction processes at both the individual and organizational levels.

Traditional research has predominantly been explored from the perspective of uncertainty, such as social uncertainty, perceptual uncertainty, action uncertainty, outcome uncertainty, etc. [14,15,16,17,18]. However, decision making by individuals in dynamic organizational contexts is often a complex process. Uncertainty only reflects one aspect of the aforementioned challenges. Therefore, a research perspective based on uncertainty/ambiguity in decision making has limitations in predicting individual efficacy in organizational change within dynamically changing environments. The concept of entropy offers a more comprehensive analytical perspective. However, describing individual behavioral outcomes in dynamically changing environments from the theoretical viewpoint of entropy remains an unresolved issue. This also renders the quantification and interpretation of entropy in psychology or organizational behavior exceptionally complex [6,19,20,21,22].

Finally, current interdisciplinary applications of entropy mainly focus on theoretical transfer and construction based on its core concepts. However, the lack of empirical research restricts the further validation and development of these theories [23]. For instance, although entropy can be used to explain organizational responses to ambiguity, complexity, and uncertainty, operationalizing these concepts in practice (at macro or micro levels), as well as examining the related processes through empirical methods, remains a challenge.

Based on the considerations above, this review firstly interprets the concept and theory of entropy and provides a comprehensive overview of the conceptual transfer of entropy across various disciplines, aiming to understand the core essence of this ultimate law governing the universe. Secondly, we delineate methods for quantifying entropy in interdisciplinary and multi-contextual backgrounds. Finally, by considering entropy a fundamental concept describing disorder or randomness within complex systems, we propose that there exists a psychological structure at the individual level for proactively controlling increases in entropy. Therefore, this article proposes an entropy-based proactive control model at the individual level and redefines “psychological entropy” accordingly. This article posits that psychological entropy reflects the meta-mindset of individuals proactively adapting to, managing, regulating, and controlling entropy changes within and outside an organization. This meta-mindset, acting as a meta-structural characteristic of the individual, not only as a meta-structure of the individual, can not only explain and predict various positive organizational behaviors but also integrates, to some extent, the behavioral outcomes of individual decisions made in situations of uncertainty, ambiguity, and complexity. For instance, the explanatory power of psychological entropy can extend the TU (tolerance of uncertainty) spectrum toward the positive. More importantly, psychological entropy can also integrate a series of psychological constructs that promote sustainable individual development, such as lean spirit, and provide theoretical support for the proposition of new constructs.

In summary, the concept of psychological entropy not only enriches our understanding of individual behaviors within organizations but also offers new perspectives and tools for management practice.

## 2. The Conceptual Development of Entropy in the Context of Various Disciplines

### 2.1. Physical Perspective

Clausius [4] first introduced the concept of entropy within the context of thermodynamics in 1865. He emphasized that, in the absence of external influences, heat always flows from a hotter body to a cooler one. However, there is always a loss in energy conversion, such as a generator never being able to achieve 100% efficiency. Clausius considered the portion of energy that could not be converted into electrical energy entropy. Thus, thermal entropy can be understood as a form of energy “residue,” that is, the energy within a heat system that cannot be utilized for work. Entropy also reflects the degree of uniform energy distribution within a system. Clausius proposed that, in a state of thermodynamic equilibrium, the distribution of energy within a system is most uniform, and there exists no cyclic process that can continuously and independently extract energy from a heat source and completely convert it into useful work [24]. At this point, the thermodynamic entropy is at its maximum. For example, when a cup of hot water and a cup of cold water are thoroughly mixed, the heat becomes uniform, with no flow of thermal or cold energy, thus reaching a state of thermodynamic equilibrium.

Subsequently, in 1877, Boltzmann proposed a probabilistic equation related to entropy (also known as the Boltzmann–Planck equation) and reinterpreted entropy from the perspective of statistical mechanics [25]. He emphasized that the entropy of a system is proportional to the number of microstates in a closed system and the probability of these microstates occurring. Consider a box filled with gas molecules; these molecules can be arranged and move in many different ways. Each specific arrangement is referred to as a “microstate”. The macrostate of this box, such as its total energy, volume, and the total number of gas molecules, is actually manifested by the collection of these microstates. Therefore, if a system has a large number of possible microstates, it becomes more difficult to accurately determine its current state, thereby increasing its uncertainty and entropy [26,27].

Subsequently, the concept of entropy was applied to explain the physical basis of living organisms [28]. Schrödinger [28] proposed that living systems are capable of reducing their own entropy by absorbing energy from their external environment, thereby maintaining their structure and function. This view serves as a complement to the Second Law of Thermodynamics, which states that the entropy of a closed system only increases. However, living systems have the ability to absorb energy from their external environment to maintain internal order. For example, plants absorb solar energy through photosynthesis, and animals obtain energy by consuming food. Both processes involve absorbing energy from the natural environment to sustain life functions, thereby helping organisms maintain or increase their internal state of order. This exemplifies the process of entropy reduction.

### 2.2. Computational Science and Information Theory Perspective

Shannon [5] introduced the concept of entropy from thermodynamics into information theory; thus, it is also known as Shannon entropy. In information theory, Shannon entropy is a measure of the novelty and uncertainty of information. The core idea is that, the greater the uncertainty of an event, the more information we obtain from it and, consequently, the higher the information entropy. For example, consider tossing a coin that has a head and a tail. When flipping the coin, the probability of each outcome is 50%, making the result uncertain. Therefore, when the coin lands, we receive information that was previously uncertain. However, if we replace this coin with a double-headed coin, the outcome of the toss is certain, and thus, the result holds no value for us. In the examples given, the toss of a regular coin has higher information entropy because its outcome is more uncertain and can provide us with new knowledge that we did not previously understand. In contrast, the toss of the double-headed coin is very certain; hence, it has low information entropy.

Wiener [29] proposed that, in cybernetic information systems, entropy represents the degree of disorder within the system [30]. He believed that, when discussing the “organization” of a system, the presence of information becomes crucial, as it forms the basis for defining and characterizing the system. From this perspective, changes in entropy are inextricably linked to changes in the organization of the system, that is, changes in its structure.

Gell-Mann emphasized that entropy is closely related to information. In fact, entropy can be seen as a measure of our degree of ignorance about unknown entities [8]. Gell-Mann viewed entropy as a measure of the uncertainty generated by an individual’s lack of understanding of the microstates within a macroscopic system. For instance, consider walking into a library rich in books. Initially, we know nothing about the variety and distribution of books in the library, not even how to find a specific book we need. At this point, we face significant uncertainty because of our ignorance of the library (the microsystem), which is a manifestation of high entropy. However, as we start using the library’s indexing system and gradually become familiar with the library’s layout and the classification of books and their specific locations, our understanding of the library improves. At this stage, by acquiring more information about the microstates (such as the distribution of books), we reduce uncertainty, which is indicative of a low entropy state.

### 2.3. Dynamic Theory Perspective

Dynamical systems are systems that evolve over time. Unlike discrete systems, whose states are fixed at specific moments, dynamical systems are chaotic and unordered, and the relationships between their elements are uncertain. In dynamical systems, entropy is often used to quantify the uncertainty of the system’s state. The higher the entropy of the system, the greater the uncertainty in predicting its future state [31]. For example, we can imagine a dynamical system as a flock of birds flying in the sky. The group flight of birds is highly complex, with each bird’s position and speed constantly changing relative to the others. These birds constitute the elements of a dynamical system, and the relationships and interactions between them are highly uncertain. Questions arise such as how information is rapidly transmitted throughout the flock, how they can change formation so swiftly, how their speeds and accelerations are distributed, and how they manage to turn together without colliding. Therefore, this complex collective behavior makes it difficult to predict the flight pattern of this flock of birds (the dynamical system) at any given moment, exemplifying high entropy.

In the theory of dynamical systems, uncertainty is often related to the initial conditions of the system, the dynamical laws of the system’s evolution, and the system’s sensitivity to initial conditions. This means that even minor changes in the initial state can lead to significant differences in the system’s behavior over time [32].

### 2.4. Understanding Entropy in the Nervous System

Entropy is used as a measure of the information-processing capacity of the nervous system [33,34,35], and it serves as a powerful tool for quantifying brain function (complexity and unpredictability) and its information-processing capabilities [36,37]. High neural entropy indicates that brain activity patterns are more complex and irregular, potentially offering greater adaptability in processing diverse information and the ability to make effective decisions in complex tasks. On the other hand, low neural entropy suggests more ordered or repetitive neural activity patterns. In such cases, the brain may not be as well-suited for processing diverse information but could be more efficient in performing certain specific, repetitive tasks [36,38].

A balanced neural entropy, which is the equilibrium between entropy and redundancy in neural activity, might represent the most efficient state for the brain to process information. This is because the brain’s capacity to process information depends not only on entropy (i.e., the diversity of information) but also on reliability, which is the balance between entropy and redundancy [39].

### 2.5. Understanding Entropy from a Psychological Perspective

Psychological entropy has been used to describe the uncertainty and disorder in an individual’s mental state [22]. For instance, conflicting beliefs, unclear self-concepts, or unresolved decision-making difficulties all signify higher psychological entropy. This is often accompanied by cognitive and emotional turmoil [40]. Research indicates that, during problem solving, when initial strategies fail, a significant increase in behavioral entropy is observed, manifesting as irregularity and unpredictability in behavior [41,42]. The increase in entropy prompts individuals to seek new strategies and solutions, marking a shift in their approach. Thus, problem solving is essentially a process of reducing chaos or, in other words, lowering psychological entropy. Once a new effective strategy is formed, behavioral patterns tend to return to a predictable, stable state of low entropy.

### 2.6. Understanding Entropy from a Sociological Perspective

In the construction of social systems, the maintenance of social order is closely linked to the criteria for classifying social roles. These criteria are diverse, encompassing aspects such as social class, educational background, abilities, and talents, collectively determining an individual’s role and status in society. When the aforementioned order and classification fail to sustain the normal functioning of social mechanisms, the social system can descend into chaos [43]. Therefore, in the field of sociology, entropy is often defined as a key indicator for measuring the degree of order, stability, and chaos/disorder within a social system, essentially reflecting the dispersion or unorganized state of social elements. In other words, entropy is also used to gauge the lack or abundance of diversity within a system [44].

Dinga, Tănăsescu, and Ionescu [45] propose that entropy and order are opportunity costs of each other and, based on the concept of social order, have developed a novel theoretical framework for social entropy. They argue that social entropy fundamentally rests on social norms and must be related to social order. Specifically, social entropy is inversely proportional to social order. A society that is orderly and adheres to rules exhibits lower social entropy. However, social entropy is not merely a representation of a society’s state of disorder. Dinga and colleagues [45] identified three core structures essential for an individual’s fit within society: self-esteem, freedom, and democracy. When these three core needs are not met within a society, it leads to a deviation from social order, resulting in increased social entropy and heightened societal chaos. In summary, their concept of social entropy is largely based on the values and demands of social justice [45].

### 2.7. Organizational System Perspective

Organizations are often conceived of as systems, typically described as collections of interconnected or interacting elements. Testa and Kier [46] suggest that a system can be characterized in three aspects. Firstly, a system needs to have a structure (form) that can be formally described. Secondly, the system must exhibit functional behavioral patterns, meaning that the behaviors among individuals are interrelated, focusing on their characteristic properties rather than the dimension of time. Thirdly, the form and function of a system are not static but change over time, which can be described as complex system fluctuations. Therefore, organizational entropy often quantifies the level of chaos or disorder within an organizational system. This disorder may arise from a combination of factors within the organization, such as decision making, communication, technology, or culture. Such a state of high entropy not only consumes resources and reduces efficiency but may also hinder an organization’s innovation and adaptability [47,48,49]. Assessing organizational entropy considers the organization’s ability to maintain a differentiated state, which is relevant for fostering the long-term sustainable development of the organization [48].

### 2.8. Entropy from the Perspective of Management

Management entropy is used to describe the chaotic and unsustainable state within an organization caused by factors such as information asymmetry, unclear objectives, inefficient workflow, and resource misallocation [5,50,51]. The greater the chaos within an organization, the higher the management entropy. Kast and Rosenzweig [50] proposed that organizational systems can import resources from their environment, that is, by maintaining a continuous flow of matter, energy, and information to achieve a dynamic equilibrium state, thereby reducing management entropy. Therefore, addressing management entropy is an inevitable challenge for every organization, rooted in organizational complexity and human diversity. Reducing management entropy not only enhances the operational efficiency of an organization but also contributes to creating a more harmonious working environment. This implies that the introduction of the concepts and methodologies of thermodynamic entropy into management is crucial, as they allow us to address management issues from new perspectives [52].

## 3. Quantification and Application of Entropy in the Context of Various Disciplines

In various academic fields, quantifying the degree of uncertainty in events involving stochastic processes is a pervasive challenge. This uncertainty often implies disorder, ambiguity, and a lack of predictability, making the prediction of stochastic processes extremely difficult. Against this backdrop, the concept of entropy becomes a key tool for understanding and quantifying the uncertainty, ambiguity, and disorder of systems. The quantification of entropy spans multiple domains, and its diversity is reflected in different types of entropy. Whether it is measuring the distribution of energy in thermodynamic systems as thermodynamic entropy, quantifying the richness of information in messages in information systems as information entropy, describing the degree of uncertainty in mental states as psychological entropy, or measuring the order and stability in social systems as social entropy, the concept of entropy provides a reliable and consistent method of quantification. This is particularly important for analyses in fields involving probability and uncertainty. Next, we will introduce a series of common and easily understandable entropy quantification concepts. This work enables us to more accurately understand and predict complex events involving stochastic processes.

### 3.1. Thermodynamic Entropy

In the Second Law of Thermodynamics, entropy is primarily used to describe energy changes and can be represented by Equation (1). Here, *dS* represents the change in entropy, T is described as the thermodynamic temperature of the system, and *dQ* is the heat change in a reversible process. Taking the process of ice melting into water as an example, when the temperature of ice decreases to 0 degrees Celsius, it melts into water. We can then calculate as follows: dS_melting_ = dQ_melting_/T. Here, Q_melting_ is the heat required for the ice to melt, and T represents the melting temperature (in Kelvin). However, this formula describes the relationship between the change in entropy of a system and the heat absorbed or released by the system in a reversible process. In practical applications, since most natural processes are irreversible, this formula is usually used for idealized analysis.

(1) $$dS=\frac{dQ}{T}$$

### 3.2. Entropy Quantification in Statistical Mechanics

In statistical mechanics, the quantification formula for entropy provides a method to understand entropy from a microscopic perspective, which can be described by Equation (2). Here, *S* represents entropy, and k_B_ is the Boltzmann constant, which provides a conversion from microscopic energy units (such as electron volts) into macroscopic energy units (such as joules). W is the number of microstates of the system, which is the number of possible microscopic arrangements of the system under given macroscopic conditions. The core idea of this formula is that entropy is directly proportional to the natural logarithm of the number of possible microstates in the system. The greater the number of microstates, the higher the entropy of the system, indicating a higher degree of disorder. We can imagine a system composed of an ideal gas (consisting of non-interacting, structureless particles). The number of microstates, W, for an ideal gas system with a given energy, volume, and number of particles can be estimated using Maxwell–Boltzmann statistics. Entropy, *S*, can then be calculated by substituting into the formula. However, although this calculation provides a powerful framework for understanding entropy from a microscopic perspective, this formula mainly explores how entropy arises from behavior at the atomic and molecular levels and typically involves complex integrals and knowledge of statistical physics, making it difficult to transfer and apply.

*S* = k_B_ ln(W) (2)

### 3.3. Information Entropy

Shannon [5] introduced the concept of entropy into information theory and proposed a method for measuring the amount of information based on the aforementioned formula, as shown in Equation (3). This formula is very important in both statistical mechanics and information theory. It indicates that entropy is the negative sum of the probabilities of all possible states multiplied by their logarithms. In information theory, it measures the uncertainty of information or the average amount of information. In statistical mechanics, it describes the uncertainty or disorder of the system’s microstates. In the formula, S represents entropy, K is a positive constant (such as K = 1), and p_i_ represents the probability of the i-th microstate. The summation is over all possible microstates. This also means that we must determine the potential probability of an event occurring in a random process as accurately as possible.

Take the result of a coin toss as an example. Assuming the coin toss is fair, ideally, the probability of getting heads or tails is 0.5 each. Applying Shannon’s entropy formula, we can calculate the entropy: *I* = −(0.5log(0.5) + 0.5log(0.5)). If we use logarithms to the base 2, substituting into the formula, we obtain *I* = 1 bit (the unit of entropy is bits). This means that, on average, each coin toss provides 1 bit of information.

Shannon entropy can also describe the richness of information. Take the string “0001000100010001…” as an example. Based on this string, we can calculate the probabilities of 0 and 1 appearing in the string. We find *P*(0) = 0.75, *P*(1) = 0.25. Still using logarithms to the base 2, we have *I* = −(0.75log0.75 + 0.25log0.25) ≈ 0.811. It is worth noting that some studies have provided more precise formulas for the value of *K*, such as depending on the length (b) of a finite alphabet, A, and considering *K* = 1/log_2_b.

(3) $$I=-K\sum_{i=i}^{n}P_{i}\;\log\;P_{i}$$

### 3.4. The Quantification and Application of Entropy in Social Science

Given the universality and stability of Shannon entropy in measuring uncertainty and complexity, it has been widely applied in the field of social sciences.

#### 3.4.1. Psychological Entropy

As mentioned earlier, psychological entropy is used to describe the uncertainty and disarray in an individual’s mental state. Hirsh, Mar, and Peterson [22], drawing on Shannon entropy, have developed a method for calculating psychological entropy. Their entropy of uncertainty model (EUM) conceptualizes an individual’s perceptual and behavioral processes as a probability distribution. The perceptual process is understood as an individual’s interpretation of sensory input based on expectations, motivations, and prior experiences. Thus, it is possible to quantify a probability distribution of potential meanings and perceptual experiences from any given sensory input. Concurrently, an individual’s potential actions also follow a probability distribution [53]. Therefore, Hirsh et al. [22] propose that the uncertainty associated with a given perceptual or behavioral experience can be quantified using Shannon entropy, as shown in Equation (4). This formula reflects the negative logarithmic sum of the probabilities of each possible outcome. For instance, in a scenario with four potential outcomes, X_1_, X_2_, X_3_, and X_4_, if the probability of one outcome is significantly higher than the others, it implies a lower level of psychological entropy. Conversely, if the probabilities of all four outcomes are evenly distributed, it indicates a higher level of psychological entropy. FeldmanHall and Shenhav [6] also suggested that this method can be used to quantify an individual’s social uncertainty.

(4) $$\mathrm{Entropy}\;=-\sum_{i=1}^{n}P\left(x_{i}\right)\;\log_{2}\;P\;\left(x_{i}\right)$$

#### 3.4.2. Organizational Entropy

To assess the sustainability of an organization, the concept of entropy can be utilized to quantify the level of understanding of the organizational system. Martínez-Berumen et al. [48] provide an approach for this. Initially, it is necessary to identify the organizational system to be evaluated. Subsequently, a range of organizational scenarios that can describe potential risk levels for the organization’s long-term sustainable development should be determined. Martínez-Berumen et al. [48] suggest considering up to 11 scenarios, ranging from Scenario 0 (indicating high risk) to Scenario 10 (indicating low risk). The next step involves identifying variables within each scenario that may contribute to uncertainty (e.g., innovation, talent, culture, leadership, structure, etc.). An assessment based on a specific scenario, such as innovation, is then conducted to obtain a probability distribution. This distribution is subsequently used in the calculation of Shannon entropy.

Martínez-Berumen et al. [48] also propose a quantitative indicator of organizational entropy: when 3/4InK < S ≤ InK, it indicates a high level of chaos within the organizational system; when 1/2InK < S ≤ 3/4InK, it suggests that the organizational system is orderly. Organizations are advised to focus on the trend of “organizational sustainability” and determine if any factors need strengthening. When 0 < S ≤ 1/2InK, the organizational system is highly orderly, where K represents the number of defined scenarios (11 in this case). Therefore, the entropy of an organizational system can be used to assess the risks faced by the organization and its long-term sustainability [48].

#### 3.4.3. Social Entropy

Social Entropy Theory (SET), proposed by Bailey [54,55], offers a framework for understanding and quantifying the disorder and uncertainty in social systems. Bailey suggests that social entropy can be assessed using a framework known as PILOTS (Bailey, 1997; Bailey, 2008). Within the PILOTS framework, society is viewed as a bounded spatial region (S), characterized by its population (P) and various informational elements such as information (I) and technology (T). These variables collectively form a complex network and, through self-organization (O), achieve a level of entropy minimization, thereby optimizing the quality of life (L).

In the PILOTS framework, the elements do not directly involve specific information at the individual level but describe the attributes of the entire society through a series of macro variables. For instance, population (P) is subdivided into individuals with immutable characteristics, including gender (G), race (R), and age (A), collectively referred to as GRA. Therefore, we can assess a system’s social entropy based on this framework. For example, to evaluate the social entropy index of city A, we can quantify social entropy by assessing the diversity and complexity of different social groups, economic activities, cultural activities, the distribution of city resources (such as education, healthcare, and housing), and the city’s response and resilience to external shocks like economic crises and natural disasters. When city A possesses strong economic stability and adaptability, it often indicates lower social entropy; conversely, an uneven distribution of resources in the city can lead to an increase in social entropy.

Additionally, Takaguchi et al. [56] proposed using the information entropy method to predict a sequence of conversations among individuals [56]. Peng and others utilized Shannon entropy to demonstrate the focus of Twitter users on different topics compared with the entire system [57]. Kulisiewicz et al. [58] suggested that entropy (calculating first-order, second-order, and third-order entropy) could be used to describe the dynamics of human communication mechanisms in social networks, which can help us observe and understand sociological processes in dynamic communities [58]. Westbury and others [59] used Shannon entropy to predict the humorous response generated by meaningless strings (non-word strings, NWs). The results showed that Shannon entropy does indeed correctly predict human judgments of NW funniness, also demonstrating that the perceived humor is a quantifiable function of how far the NWs are from being words.

In summary, the current approach to entropy calculation across various disciplines is primarily based on the concept of information entropy. This involves striving to ascertain the latent probability of a specific event occurring within a random process to facilitate the computation of entropy. However, quantifying the latent probability of an event’s occurrence is undoubtedly not a trivial task. This challenge hinders the quantification and calculation of entropy in certain disciplinary contexts. Consequently, integrating different disciplinary characteristics to adopt varied methods for entropy quantification is a complex and nuanced process. It necessitates a profound understanding of the nature of entropy and the inherent uncertainties involved.

## 4. How Do Individuals Cope with and Manage Uncertainty in Entropy Increase?

As mentioned, entropy is a fundamental concept used to describe ambiguity, disorder, complexity, and uncertainty within complex systems, and individuals possess an innate ability to reduce entropy in such environments. However, why can some individuals effectively cope with increasing entropy to achieve sustainable development while others are gradually “consumed” by it? We believe that the difference in outcomes depends on the individual’s ability to control entropy. In traditional research, psychologists and management scientists have attempted to answer this question by studying “uncertainty”. Although the disorder, complexity, and randomness inherent to entropy can trigger an individual’s perception of uncertainty [60], fundamentally, we consider the process of controlling and managing uncertainty, whether originating internally or externally, to be part of entropy management.

In the traditional field of uncertainty research, psychology offers integrative concepts and mid-level generalizations [18]. Uncertainty implies a lack of reliability, credibility, or adequacy [61]. Information characterized by uncertainty can lead to self-doubt in individuals and have a detrimental impact on their thoughts and behaviors [62]. Therefore, at the individual level, social (organizational) behavior has a critical and potent motivator, namely, the desire to reduce uncertainty [6].

In many behavioral theories, psychological uncertainty is considered an important mediator in human responses to unknown outcomes [63]. Psychological uncertainty is defined as a psychological structure that includes a variety of potential positive or negative psychological effects [64,65]. However, in most cases, uncertainty is seen as a negative influence, for example, inducing worry, anxiety sensitivity, fear of negative evaluation, perceptions of vulnerability, and avoidance of decision making in individuals [15,17]. Faced with these effects, individuals adopt different strategies and responses to mitigate the negative effects of uncertainty. Some individuals tend to use passive coping mechanisms, such as attention diversion to ignore uncertainty, thereby achieving emotional regulation [15], while others, although bravely acknowledging and confronting uncertainty, experience reduced action efficacy because of the resulting fear, anxiety, and disempowerment, accompanied by emotional dysregulation [66,67]. Further, some research indicates that an individual’s experiential permeability (EP) is a key factor determining whether they can positively cope with uncertain situations. In other words, if one person’s knowledge is complete, it is difficult for them to experience uncertainty [68], which, in turn, prompts them to discover and benefit from the positive effects hidden in uncertainty [69]. Therefore, depending on the specific environment, individuals exhibit different responses to uncertainty, depending on individual personality traits and differences in strategies for coping with uncertainty [70].

In the realm of decision-making research, Herbert A. Simon, as early as in his work referenced as [71], delved into the behavioral responses of decision-makers when faced with uncertainty. He suggested that decision-makers should be viewed as having bounded rationality, largely because individuals cannot know all alternatives, hold uncertain attitudes toward exogenous events, and lack the ability to estimate outcomes. Kahneman and Tversky proposed that individuals’ rules of perception and intuitive judgments significantly affect their decision making in the face of uncertainty. They explored how individuals use heuristics in uncertain situations and the biases they are prone to in various judgment tasks, such as predictions and evaluations of evidence [72,73,74]. They also studied individuals’ loss aversion in riskless choices [75,76] and how estimates of the probability of uncertain outcomes in prospects become a determining factor in decision making (prospect theory) [76]. Subsequently, Kahneman [18] proposed that intuition and reasoning are alternative methods of problem solving and described the role of prototype heuristics in uncertain decision-making tasks. In recent years, FeldmanHall and Shenhav [6] combined Bayesian thinking to propose three methods of reducing uncertainty: automatic inference, controlled inference, and social learning. Moreover, emotional regulation methods also determine individuals’ decision-making responses in situations of uncertainty (including adaptive or maladaptive strategies [14,16,66,77]).

Although the aforementioned studies focus on exploring what kind of irrational behaviors individuals exhibit in scenarios of uncertainty or what cognitive strategies and emotional regulation methods they can use to reduce uncertainty, they overlook the important capacity of individuals to consider prognostic activity as a meaningful variable, as well as the related goal setting and thinking processes [64]. In other words, discussions of uncertainty in decision-making contexts are mainly conducted within the frameworks of cognitive psychology and organizational decision research, lacking an examination of differences between decision-makers and research into related traits and abilities.

Therefore, the academic community has begun to focus on the important role of an individual’s psychological state/traits in influencing their decisions and responses to uncertainty. Since the 1990s, some scholars have identified the difficulty in handling uncertainty as a distinguishable personality trait. It is really a predisposition [78,79,80]. Consequently, many studies turn to exploring individual (in)tolerance of uncertainty, which has received more extensive exploration within the discipline of clinical psychology [81,82]

Intolerance of uncertainty (IU) refers to the negative emotions or beliefs triggered in individuals because of the perception of lacking significant, critical, or sufficient information [83,84]. This tendency toward negative responses may manifest at the emotional, cognitive, and behavioral levels and is maintained by related perceptions of uncertainty [85]. Tolerance to uncertainty tends to describe an individual’s emotional response to their orientation toward an undetermined future [86]. Individuals with higher levels of IU view uncertainty as a source of stress, discomfort, fear, and conflict [87,88,89] and find it difficult to tolerate aversive experiences related to uncertainty [90]. Research shows that higher levels of IU are transdiagnostic risk factors for many clinical disorders, including anxiety, depression, obsessive–compulsive disorder, and eating disorders [91,92].

Tolerance and intolerance for uncertainty are key variables in the overall system of individual choices and decision-making regulation under conditions of uncertainty. The concept of tolerance for uncertainty proposed by Frenkel-Brunswik [93] is subject to substantial definitional heterogeneity. Although initially IU and UT were studied as traits in the fields of cognition and personality, over time, IU and UT have gone from being viewed as being two poles of the same conceptual continuum to being partly independent constructs and dimensions of personality [94]. Tolerance of uncertainty (TU) emphasizes “tolerance”. Hillen et al. [15], in their study, note that to “tolerate” means ”to allow” (something that is bad, unpleasant, etc.) to exist, ”happen or be done”, or “to experience (something harmful or unpleasant) without being harmed”. This means that, in TU, the most an individual can do is to remain unaffected by negative events. Furthermore, where does the boundary of TU begin and end? In individual responses to uncertain situations, which responses should be considered to constitute the phenomenon of TU itself rather than just being produced by TU is also a matter of debate.

Furthermore, there is debate over whether TU and IU can represent a stable personality trait that predisposes individuals to specific psychological responses [70,79,95]. Some of the literature suggests that TU is predominantly a psychological trait [79]; thus, these studies typically view TU as a measurable and stable construct and often omit exploring context-specific manifestations of uncertainty [15,96]. Where TU is explored as a modifiable state, the state of TU is influenced by either contextual or situational factors that may alter the individual’s TU condition [70,80]. Hillen et al. [70] developed a contemporary and comprehensive integrative model of uncertainty tolerance (IMUT) and suggested that exploring TU as either a trait or a state is appropriate.

In summary, Simon and Kahneman primarily studied individual decision-making behavior in uncertain situations in a cognitive framework [18,71]. Furthermore, both IU and TU reflect the emotional response dimension of those experiencing uncertainty, embodying an individual’s anticipation and interpretation of future outcomes under uncertain conditions. Therefore, although previous research has explored individual coping strategies and behavioral responses from the perspective of uncertainty, it does not explain which personality traits and coping methods enable individuals to proactively face uncertainty in organizational development/change. From this perspective, research on organizational entropy change can provide clearer, more comprehensive answers.

On the other hand, Hirsh et al. proposed a concept of psychological entropy at the individual level, used to describe the uncertainty and chaos of an individual’s mental state [22]. However, this research suggests that an increase in entropy is a sign prompting individuals to seek new strategies and solutions (problem solving). Like the aforementioned uncertainty research, it does not address under what conditions people seek more rather than less uncertainty, nor whether individual differences in uncertainty seeking reflect a positive feeling toward uncertainty itself or a desire for information and/or solutions to aversion to uncertainty.

Therefore, this paper posits that entropy change occurs at various stages of life and societal development, with entropy reduction being an innate tendency of living organisms, determining orderly individual development. We attempt to propose a meta-mindset at the individual level by analyzing and understanding the theoretical content of entropy, combining existing entropy research in psychology, and building on perceptual research of uncertainty. Upon redefining psychological entropy, we propose an active entropy control model. Through this model, we aim to deepen the understanding of how individuals with certain mental models can better face disorder, ambiguity, complexity, and uncertainty in situations such as organizational change and possess the ability to predict positive organizational outcomes.

## 5. Entropy-Based Proactive Control Model

Entropy and energy form the foundation of all natural processes, including human activities. Despite the fact that thermodynamics has been established for over a century and a half, no amount of technological advancement or theoretical innovation has been able to undermine its principles. This holds true even for the forward-thinking and revolutionary quantum theory [97]. Many physicists unanimously agree that the most convincing and encompassing laws in physics are embodied within the laws of thermodynamics. All interacting natural forces and processes adhere to the laws of energy and entropy. Therefore, entropy is not only a focal point for interdisciplinary unified knowledge but, more importantly, it can serve as a focal point for the interdisciplinary unification of knowledge and, to some extent, embodies characteristics of the “Grand Unified Theory” that Einstein pursued throughout his life [98]. From the perspective of life development, the entropy reduction phenomenon inherent to human biological instincts may have the capacity to generalize entropy control tendencies and personality traits in organizational management contexts. This implies that individuals within organizations have an inherent motivation to actively reduce uncertainty (entropy reduction). Han et al. [60] regard uncertainty as a fundamental metacognitive state consisting of the conscious awareness of ignorance [99]. It arises from unconscious brain mechanisms, functioning independently of rational thought [100]. Therefore, we attempt to propose a meta-concept from the perspective of entropy to describe the proactive control of disorder and uncertainty within a system by individuals. This includes the mindset of individuals actively controlling entropy in organizational contexts and explains how individuals can proactively deal with uncertainty to achieve organizational success. We believe that psychological entropy has the rich connotation of integrating various organizational management constructs and can broadly predict and explain a variety of behaviors of individuals within organizations. To achieve this, it is imperative to first delineate and understand several key concepts.

Firstly, it is essential to properly understand increases and reductions in entropy. In the field of psychology, Hirsh et al. [22] proposed the entropy uncertainty model (EUM). This model conceptualizes the realms of perceptual and behavioral uncertainty as probability distributions, revealing how individuals interpret sensory input based on expectations, motivations, and past experiences. The EUM emphasizes that individuals strive to reduce uncertainty to a manageable level, thereby alleviating psychological discomfort caused by the uncertainty of perception and behavior. However, this model appears to reflect the essence of determinism, exemplified by Newtonian thought: order dominates everything. It (the model) underscores the individual’s motivation to actively seek to change the state of disorder [13,101]. However, humans are complex organisms, and maintaining equilibrium can involve two distinct types of activities: “preventing entropy increase” and “facilitating entropy reduction”. For example, in physical exercise, human muscle cells accelerate the breakdown of carbohydrates and fats to generate motion and energy. This process produces waste. If this waste is not expelled from the body through an open system, it is impossible to “prevent entropy increase”, leading to the collapse of the organic system. Simultaneously, physical exercise is also a process of ”facilitating entropy reduction”. Through exercise, the muscle structure of the human body becomes more ordered and efficient. Muscle cells and the nervous system gradually adapt through repeated movement training, enhancing the coordination and efficiency of movement. Hence, the continuous attainment of coordination and proficiency in muscle tissues represents an individual’s effort to achieve entropy reduction. The life system encompasses both “preventing entropy increase” and “facilitating entropy reduction”, two complementary processes [102].

Secondly, dissipative structures, open systems, and entropy reduction are critical to the continuation of life. Prigogine introduced the concept of dissipative structures based on Bénard convection experiments, thereby revealing how structures, organizations, and order emerge in the face of anomalies, turbulence, disorder, and dissipation [103,104]. A dissipative structure refers to a complex and ordered structure that spontaneously forms in an open system far from thermodynamic equilibrium when the system reaches certain critical conditions through the exchange of matter and energy with its surroundings [105]. In fact, as hypothesized in thermodynamics, in an absolutely closed system, entropy tends toward infinity because the system becomes increasingly disordered and incapable of coping. However, both life systems and organizational systems formed from numerous life systems are open systems composed of dissipative structures [106]. They continuously exchange with the external environment, allowing the system to adopt measures, such as actively taking interventions (like energy) from the external environment to reorganize the internal disorder, thereby achieving entropy reduction. In other words, to survive and sustain development, it is first necessary to maintain an open state. The system provides conditions for the evolution of its complexity by constantly resisting, absorbing, or even transforming disorder. It can be said that, for a system’s order and organization to sustain development, it must possess the capacity to tolerate, utilize, and proactively regulate states of disorder [107].

Thirdly, emergence often occurs in the interplay between order and disorder, and it is key in advancing order and achieving entropy reduction within open systems [108]. Emergence is a unique phenomenon in open systems, referring to new and holistic properties or behaviors that arise from the interactions of the system’s various parts. These properties do not exist within the individual components of the system. Emergence can be understood from the perspective of the transition between order and disorder. Diversity embodies disorder, and disorder generates diversity; unity represents order, and thus, the unification of diversity is emergence. For instance, the large-scale aggregation of mass in the universe to form black holes is an example of emergence—a new structure. Similarly, a group of musicians playing randomly is akin to chaos and disorder, like grains of sand being scattered. However, under the conductor’s unified organization, they form an organic harmony, playing the same piece in a structured and regulated manner, resulting in a high degree of order—this is emergence. In this process, the active organization of the conductor (akin to doing work) is crucial. Hence, it is evident that emergence (the unification of diversity) is a core characteristic of the continuous development of an organization.

In summary, we believe that achieving the sustainable survival and development of organizations requires individuals to actively exercise their agency. This necessitates that they not only guard against the emergence of disorder but also respond to disorder in a manner consistent with the organization’s survival needs. This ensures that the system neither disintegrates because of disorder nor becomes rigid because of order. Ultimately, by managing both order and disorder, continuous emergence can be achieved [109]. Based on this premise, this paper introduces an entropy-based proactive control model.

Indeed, we believe that existing discussions based on entropy predominantly focus on the perspective of information entropy, that is, the disorder and chaos of the system, to explain specific issues. However, a precise understanding of the Second Law of Thermodynamics and the essence of entropy aids in better explaining the complex evolutionary processes of life systems and organizational systems. This paper attempts to draw upon certain aspects of fundamentalism and meticulously executes its theoretical transition based on the core essence of “entropy change”. First, entropy possesses the following characteristics [45,110,111,112,113]:

1. Entropy is a concept of maximum generality, applicable to any of the three worlds in Popper’s framework.
2. Entropy can be formalized as a state variable, a state function, or a state vector.
3. The magnitude of entropy’s change depends solely on the initial and final states.
4. Entropy is a parameter, with its magnitude inversely proportional to the degree of order.
5. Entropy is non-static, meaning that, in a closed system, entropy inevitably and permanently increases.
6. Global entropy (i.e., the entropy within a closed system) is irreversible.
7. Entropy is a macroscopic variable determined through the integration of microstate simulations, and it exhibits macroscopic irreversibility.
8. According to statistical thermodynamics formulas, entropy is a statistical quantity.
9. Entropy is an additive variable.

After a detailed clarification of the fundamental characteristics of entropy, we can now examine the entropy change process in the context of individuals and organizations based on the four core implications of entropy change:

(a) Entropy reduction occurs in open systems/dissipative systems.
(b) The higher the concentration of high-quality energy, the lower the entropy.
(c) When a system is in equilibrium, energy is most dispersed (the most configurations), resulting in higher entropy.
(d) The complexity of critical states/self-organization states is highest, leading to higher entropy.

Building upon these principles, this paper, based on an accurate grasp of entropy change, proposes a model for explaining how organizations can achieve sustainable development by addressing disorder/uncertainty in transformational contexts, namely, through an entropy-based proactive control model. This model integrates four core concepts from psychology and organizational behavior, each corresponding to one of the four key connotations of entropy change, including learning orientation, goal orientation, change orientation, and risk taking, thereby playing a descriptive and predictive role in how individuals within organizations cope with the process of increasing entropy.

We believe that psychological entropy reflects the meta-mindset of individuals in proactively adapting, managing, regulating, and controlling the ”entropy changes” within and outside an organization. Psychological entropy, through the adjustment of individual agency, drives the organizational system to break relative equilibrium, enhance organizational functional complexity, and achieve dynamic stability and high-level development at the organizational level.

Specifically, individuals within the organization exhibit a strong ability for continuous learning and active adaptation to new information, strategies, and methods. This meta-mindset motivates them to actively set strategic goals, continually advance in uncertain situations, and courageously take risks to facilitate adaptive evolution and innovation within the organizational system. When individuals possess a high level of psychological entropy, it often signifies their strong abilities in entropy adaptation and control. Next, we will introduce each of the four components of the entropy-based proactive control model.

### 5.1. Dissipative System and Learning Orientation

Prigogine [104] first introduced and detailed dissipative systems, which are the subject of research on how open systems interact with their environments [114]. As mentioned earlier, the entropy of isolated and closed systems only increases and never decreases. However, dissipative systems break away from the traditional closed system model, providing a framework for understanding how open systems generate order and structure from non-equilibrium conditions [10].

Unlike traditional closed systems, open systems can exchange energy, matter, and information with the external environment, allowing them to maintain non-equilibrium states. These states are variable and dynamic and can generate new ordered structures, known as dissipative structures. Prigogine and Stengers [115] further extended this “entropy reduction” framework to biological organisms, where individuals can also be viewed as open dissipative systems. Therefore, to maintain their stability and organizational structure, internal entropy must be effectively transferred to the external environment. This provides us with an important insight, in that individuals need to proactively construct an efficient dissipative system by continuously exchanging energy, matter, and information to maintain non-equilibrium states and generate new ordered structures, thus better managing changes in entropy.

Based on the fundamental properties of entropy and dissipative systems, we propose that psychological entropy should include the core component of learning orientation.

Learning orientation is a set of values that influences the degree to which proactive learning occurs [116]. Individuals with a learning orientation often possess an open mindset and a commitment to learning. They do not confine themselves to existing and fixed thought patterns; instead, they proactively embrace new knowledge and new experiences. Through the exchange of information between new and old knowledge, they break through tradition and generate creative thinking [116].

Furthermore, learning orientation encourages individuals to respond and adapt quickly to organizational contexts. It equips them with the ability to continuously enhance their competitive advantage within the organization through knowledge sharing, exchange, and absorption [117]. Previous research on individual responses to uncertainty has suggested that an individual’s tolerance of uncertainty is highly correlated with openness (i.e., experiential permeability [69,118,119]). Additionally, uncertainty reduction theory also posits that individuals have a motivation to actively acquire external information and resources to reduce uncertainty [120], particularly in continuously developing and changing scenarios, where information acquisition is crucial. Therefore, we propose that learning orientation is a crucial capability for individuals to proactively adapt to the uncertain organizational environment, making them a form of dissipative structure.

We believe that cognitive–behavioral systems fundamentally adhere to the same basic principles as other dissipative systems, and the sustainability of cognitive–behavioral systems depends on the ability of dissipative systems to reduce entropy. Individuals with a high learning orientation are precisely those who can promote knowledge absorption and information exchange both internally and externally through open thinking and a commitment to learning. They reshape their neural connections and activity patterns to respond to environmental challenges and uncertainties, ultimately maintaining their functionality and stability [121,122], thereby promoting entropy reduction. In conclusion, we believe that the entropy-based proactive control model should include an individual-level dissipative process, namely, learning orientation.

### 5.2. Concentrated Energy and Goal Orientation

According to the principles of thermodynamics, entropy (a measure of disorder) in a closed system is always increasing. However, based on the second core feature of entropy change, in an open system, the more concentrated the high-quality energy, the lower the entropy [123]. Prigogine [124] and Doll [125] also proposed that, as a system is injected with increasing amounts of energy, it will “transform” into a state far from thermodynamic equilibrium. Similarly, the activities of individuals/organizations should also be goal-oriented, focusing energy and effort more effectively, thereby achieving a more efficient “transformation,” i.e., entropy reduction. We believe that goal orientation offers a method of realizing this approach.

Goals represent the specific cognitive representation of an individual’s desires and can also be understood as a state of intentional behavior guidance [126]. For the realization of a desire, individuals must set clear goals to gather focused energy toward the goal until it is ultimately achieved [127]. In organizational behavior research, goal orientation is often seen as a stable, trait-like characteristic that varies among individuals [128]. Goal orientation is also typically conceptualized as a personality disposition and measured as a trait-like individual difference variable. From the perspective of personality traits, goal orientation can initiate purposeful goal striving [129]. Goals often influence an individual’s perception and behavior by affecting the processing of goal-relevant information and the selection of behaviors [130,131,132]. Previous studies have shown that the higher an individual’s intolerance of uncertainty (IU) is, the lower their level of self-control becomes, making it more difficult to anticipate future scenarios. Consequently, this leads to a lack of capacity to facilitate goal setting from a future time perspective [133]. Consequently, when individuals possess a high level of psychological entropy, the included goal orientation enables individuals to focus their activities more sharply, with greater purpose, and with higher efficiency in terms of survival.

Individual differences in the proactive selection, determination, and pursuit of future goals directly influence organizational achievement [129]. When individuals are in a “goal-deficient” state, such as failing to set clear goals or when existing goals are abandoned without new goals to replace them, they experience high levels of entropy and wastage regarding information resources [134]. In such instances, a clear goal framework as a behavioral guide is crucial. Although the process of establishing goals can introduce some uncertainty in the short term, as it requires the mobilization of cognitive resources to identify new potential behavioral paths, when a new decision is perceived as promoting the achievement of goals, it becomes the dominant choice for the individual, thereby reducing entropy to a level lower than before.

Moreover, when individuals are in a state of “goal masking”, where their established goals are disrupted or obscured by uncertain organizational/environmental cues, they also experience heightened decision ambiguity and behavioral uncertainty, resulting in a high entropy state. In such situations, individuals with high goal orientation can proactively break down the currently obscured goals into a series of more specific sub-goals using dynamic programming techniques [135,136]. Subsequently, by tapping into a wealth of information resources (stemming from a learning orientation), they direct their focus toward these more specific sub-goals, thereby gaining localized and focused psychological energy. In summary, individuals can proactively manage and regulate uncertainty within an organization through goal orientation, adapting to the continuously changing organizational context. Therefore, we have incorporated goal orientation into the entropy-based proactive control model presented in this paper. We believe that having goal orientation enables individuals to proactively set or break down goals based on real situations, thereby stimulating stronger motivational drives. This leads to the acquisition of high-quality energy directed toward behaviors, facilitating the achievement of goals and the attainment of a state of entropy reduction.

### 5.3. Thermodynamic Equilibrium and Change Orientation

According to the third core feature of entropy, when a system is in equilibrium, it implies a more dispersed energy distribution, with the most configurations and the highest entropy, and the system undergoes no macroscopic changes [137]. To achieve entropy reduction, it is necessary to break this state of equilibrium, which is the process of emergence. Non-equilibrium states and nonlinear interactions within these states act as catalysts (key mechanisms) for emergence. Metaphorically, emergence refers to a phenomenon that exists in one dimension but not in another. Under non-equilibrium conditions, systems far from stable states may, through interactions between components, lead to the emergence of new structures and patterns [10]. Therefore, creating and maintaining a dis-equilibrium state in an organization is a requisite aspect of emergence [125,138,139,140]. Studies show that emergence is often triggered by “unconventional” activities/events (events occurring “outside the norm”), pushing the system into a highly dynamic state [125,141]. Lichtenstein and Plowman [21], analyzing three empirical studies on emergence within organizations [142,143,144], identified four constructs of emergence at successive organizational levels. These include a dis-equilibrium state, amplifying actions, recombination/self-organization, and stabilizing feedback. They argue that these four structures are necessary conditions for the emergence of a new order but not sufficient conditions. Dis-equilibrium could be caused by the proactive pursuit of new opportunities, threats, or crises from within the environment/system, or fluctuations that alter the entire organizational system. From this perspective, this paper proposes that individuals and organizations seeking high-quality evolution and development need to maintain and manage non-equilibrium states within the system, constantly driving emergence through changes. In other words, in an organizational context, individuals need to possess a “change orientation” trait. Therefore, we propose that change orientation constitutes a core component of our entropy-based proactive control model.

Organizational change refers to the change (reform) in an organization from its current state to a more optimized form [145]. The result of this is emergence within the organization. By definition, we find that change and emergence have many conceptual similarities; therefore, we believe that change orientation is a crucial factor in driving organizational emergence. Individuals with higher change orientation tend to exhibit greater adaptability [146], innovativeness [147], and an active approach to managing change [148]. Specifically, change orientation prompts individuals to proactively seek and drive systemic changes, regulate themselves to adapt to disorderly situations, and even proactively explore new possibilities from disorder [149,150,151]. Therefore, we incorporate change orientation into the entropy-based proactive control model proposed in this article. We believe that individuals with a change orientation can proactively embrace and manage change. In the context of change, individuals demonstrate greater adaptability, innovativeness, and openness by strengthening the clarity of their cognitive maps and goal structures, ultimately leading to the “process of emergence”.

### 5.4. Criticality and Risk Taking

According to the fourth major feature of entropy change, when a system is in a state of criticality/self-organized criticality, it exhibits the highest level of complexity and can rapidly evolve into new patterns. Criticality is seen as a kind of edge structure, which is neither completely ordered nor completely disordered. Emergence often occurs in states of criticality [152].

Criticality is often used to describe the critical points of phase transitions [153]. A phase transition describes the process of a material transforming between different states of matter. When the nature of the dominant feedback in a system changes, a phase transition occurs. Phase transitions are ubiquitous in nature and society, such as the transformation between ice and water or the succession of historical dynasties.

The characteristics of a system at its critical point are especially complex, manifested in the uncertainties of phase transitions, incompleteness of information, and nonlinear interrelationships between elements [154]. For instance, in a sustained 0-degree Celsius environment, whether water freezes or ice melts is uncertain. In organizational management, even planned organizational changes often have randomness and uncertainty in their direction and outcomes [155,156], meaning that organizational change essentially involves risk. When organizations are in a critical state, they exhibit characteristics such as complexity, disorder, and uncertainty. In this context, an individual’s organizational behavior often involves significant risks. Therefore, how one balances anticipated returns and risks will determine their behavioral performance in change scenarios [157]. Risk taking is an important form of human behavior, long used to explain the adaptability of human actions and the rationality behind them [158]. Risk taking is defined as engagement in behaviors that are associated with some probability of undesirable results [159]. Burnes et al. [160] proposed that the pursuit of a goal-directed option, which could result in multiple outcomes including some that are undesirable or potentially hazardous, should be considered an instance of individual’s risk-taking behavior [161]. Theories about risk taking can be broadly categorized into three types: the first type explains which personality traits frequently lead to risk taking [162]; the second type often explains the differences between risk seeking and risk aversion (prospect theory [163]); and the third type explains why some individuals take risks only in specific situations because they value and believe in success in those scenarios [158,164].

Risk taking is a multi-dimensional concept, and its outcomes are not always positively oriented. Zinn [165] proposed that risk taking includes key dimensions like motivation, control, reflexivity, and developing and protecting identity, which together influence the likelihood of an individual engaging in risk taking. It is noteworthy that risk taking can either be adaptive or maladaptive. When the benefits of certain activities are far outweighed by the potential harm, it is maladaptive. Conversely, it is adaptive as long as the opposite holds true [158]. Individuals can adapt successfully by systematically pursuing certain risks while avoiding others [166,167]. Therefore, we believe that individuals in an organization’s “critical state” need proactive risk taking to achieve a positive phase transition [168]. This prompts individuals to take initiative when facing uncertainty and potential negative consequences, actively responding to challenges.

Therefore, we include risk taking as the last core component of psychological entropy in our entropy-based proactive control model. We believe that individuals with a higher propensity for risk taking can fully assess and undertake risks in the uncertain environment of organizational change and hold the belief of “risk as value”. They engage in risk taking to protect or regain control over the organization, thus driving the organization’s critical state toward a desired positive direction [169,170]. Risk taking drives individuals to proactively adapt to situational changes, confront the organization’s critical state, strive to balance order and disorder to maintain the overall structure and function, and ultimately achieve a positive organizational phase transition, thereby realizing entropy reduction.

In summary, psychological entropy is a meta-mindset that reflects an individual’s proactive adaptation, management, regulation, and control of ”entropy changes” within and outside an organization. The entropy-based proactive control model proposed in this article comprises four components: learning orientation, goal orientation, change orientation, and risk taking. We have no intention of redefining the meaning of these four concepts but rather, based on the characteristics of entropy change and referencing the construction process of psychological capital [171], to conceptually integrate individual capabilities and tendencies for entropy control in change situations. We then propose a meta-concept that is richer in content and more broadly applicable. These four components constitute a high-order structure that can predict an individual’s ability to proactively control entropy. They are sequential in time and together constitute an active, dynamic process of entropy management (as shown in Table 1). This theory not only explains how individuals within an organization can promote high-quality development by actively regulating, controlling, and adapting to uncertainties but its rich content may also integrate existing constructs in organizational behavior.

## 6. Future Directions in Organizational Psychology of Entropy Research

The Second Law of Thermodynamics has been widely described as ”one of the deepest and most perfect laws in physics” [172]. In this law, entropy plays a central role and has played a crucial role in interdisciplinary transfer and application. This is because entropy provides a framework for understanding and quantifying disorder and uncertainty in systems. However, the application and expression of entropy in different disciplines are influenced by the attributes of each discipline, and interdisciplinary research approaches also add complexity and disorder to knowledge [173]. For example, scholars often introduce too many subjective descriptions when attempting to transfer knowledge from another discipline to the field they are studying. This is due to the fact that different scholars’ understandings of the same concept are influenced by their interpretative frameworks and knowledge backgrounds, which can lead to contradictory concept definitions [174]. Indeed, when introducing interdisciplinary concepts, regardless of the field, high entropy and uncertainty factors are introduced. Under the guidance of this high-entropy research approach, many authoritative but conflicting views have emerged [174,175]. This further exacerbates confusion and disorder, and already high knowledge entropy grows more rampant. Therefore, the authors hope that, through this article, more scholars can awaken to a comprehensive understanding and deep reflection on this “dominant” concept in order to achieve a comprehensive assessment of its interdisciplinary impact and practical applications. Entropy and energy have universal significance in different disciplines, making them universally valuable in all disciplines [176]. Thus, when we attempt to explain the complex world through the concept of entropy with interdisciplinary common values, it may help reduce knowledge confusion.

This article aims to integrate and unify the concept of entropy with organizational psychology based on a systematic analysis of entropy and to accurately grasp the essence of entropy. First, this article, based on the core characteristics of entropy, outlines the four major attributes of entropy change [45,110,111,112]. Subsequently, we propose a meta-mindset, namely, psychological entropy, which may have the ability to integrate multiple organizational or psychological concepts, to explore a possible entropy control mechanism, similar to a “Maxwell’s demon”, that can drive individuals to proactively achieve entropy reduction [177]. We construct the entropy-based proactive control model from a dynamic perspective—which can simulate and predict how individuals manage and control entropy in organizational environments and proactively respond to uncertainties brought about by organizational changes—in order to achieve high-quality sustainable development at both the individual and organizational levels.

**Figure 1:**
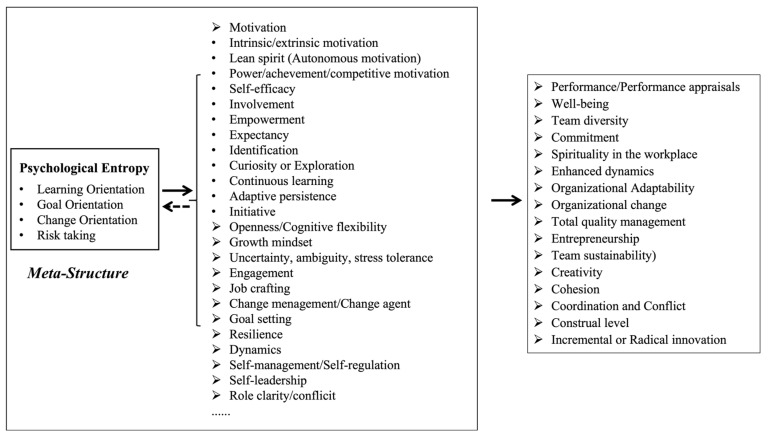
Correlation variables of psychological entropy.

More specifically, our entropy-based proactive control model encompasses four dimensions: learning orientation, goal orientation, change orientation, and risk taking. Firstly, individuals with a learning orientation are perceived as dissipative systems, equipped with the ability to proactively absorb new knowledge and execute information exchange. The essence of learning lies in guiding actions. Here, a higher level of goal orientation becomes vitally important. It enables individuals to proactively induct and organize information resources, thereby enhancing the efficiency of cognitive interpretive frameworks in utilizing information (entropy reduction). During this process, goals provide motivation for individual behavior. Moreover, high-quality organizational development necessitates individuals with a strong change orientation. Change orientation encourages individuals to proactively adapt to, manage, and regulate changing circumstances (entropy reduction) with change goals in mind, ultimately leading to emergence. However, organizational change often results in a state of organizational criticality. Criticality implies complexity and disorder. Therefore, risk taking enables individuals to bravely confront the risks associated with organizational phase transitions, proactively adapting to situational changes to address the challenges of criticality, thus fostering high-quality organizational development. The process of regulating psychological entropy is a prerequisite for survival and sustained development, and it is a key factor in individuals actively adapting to organizational environments, optimizing decisions, and facilitating personal growth. In this process, psychological entropy and its four important dimensions can integrate and explain the behavioral responses of decision-makers in uncertain scenarios to some extent. Moreover, although the entropy-based proactive control model only includes four dimensions, we believe that the rich connotation of psychological entropy is sufficient to integrate more variables of organizational behavior and to predict and explain a series of potential outcome variables (as shown in Figure 1). For example, psychological entropy reflects an individual’s ability and tendency to actively control uncertainty and thus has broad predictive utility and integration capacity for other organizational management variables that can reduce uncertainty, such as lean spirit (reflecting an individual’s autonomous motivation to reduce resource wastage, improve work efficiency, and continuously enhance work quality). This awaits further empirical research for substantiation.

Although this article proposes the concept of psychological entropy and its four dimensions, it does not delve into the methods of measuring psychological entropy. Clearly, when we rely on the concept of entropy to create a new construct in organizational behavior studies, traditional scale development and self-reporting can be utilized for measurement [178,179]. This is a highly stable and reliable quantitative path and can even be used to verify whether the proposal of these four dimensions has statistical justification. Therefore, in the future, this method can be used to develop a set of measurement tools.

Furthermore, Shannon’s quantitative formula for entropy also provides us with a series of new ideas for quantification [5,22]. In previous research, predictions about the probability of an event occurring were mainly through variance in the predictions themselves (known as risk when predicting possible rewards) [180,181], but FeldmanHall and Shenhav [6] suggested using the Shannon entropy concept to compute (non)social uncertainty [5] and quantifying total uncertainty (nonsocial + social) based on the conditional entropy method. As mentioned above, the calculation of Shannon entropy is based on the probability of an event occurring within a system. Similarly, we can quantify the entropy of a variable within an organization based on the concept of probability. Taking learning orientation as an example, suppose we conducted a survey among 100 employees, from which we obtained a dataset of scores for each employee in the dimension of learning orientation.

For instance, using the Likert scale method, we can obtain a dataset consisting of a series of continuous data. This paper will provide two measurement approaches. Firstly, we can calculate the probability distribution of participants on a 1–7-point rating scale and then input this into the aforementioned formula to calculate the corresponding entropy value. In addition, we can set a categorization threshold to classify this continuous dataset into “high” and “low” categories. The threshold can be set based on data distribution and research purposes, such as percentiles, median, mean, standard deviation, natural data segmentation points, the extreme grouping method (27%), etc. Zou et al. also proposed a sampling-based threshold auto-tuning method (machine learning) for imbalanced classification [182]. Suppose, according to the classification threshold, we distinguish 60 people with higher learning orientation and 40 with lower learning orientation; we can then calculate their probabilities, which are 0.6 and 0.4, respectively, and subsequently use them in the Shannon entropy formula for calculation. We can use this method to calculate the entropy levels of learning orientation, goal orientation, change orientation, and risk taking. Since these four variables are conceptually independent and together constitute a higher-order concept, this paper proposes a possible calculation method: joint entropy.

Joint entropy can calculate the total entropy of multiple variables and provide information about the uncertainty of the entire system [5]. Suppose there are two events, X and Y, and let *P*(I, j) be the probability of the first event, i, and the second event, j, occurring simultaneously. Then, the probability of joint entropy can be represented by Equation (5). Since the four dimensions of psychological entropy are conceptually independent, we can calculate by adding the information entropy of each and then subtracting the interference of mutual information [183]. Let us denote learning orientation, goal orientation, change orientation, and risk taking as A, B, C, and D, respectively. Therefore, the calculation of psychological entropy is conducted as follows: H(A, B, C, D) = H(A) + H(B) + H(C) + H(D) − *I*(A, B) − *I*(A, C) − *I*(A, D) − *I*(B, C) − *I*(B, D) − *I*(C, D). Here, H(A), H(B), H(C), and H(D) represent the entropy of the four dimensions respectively, while “I” denotes the mutual information between variables.

Mutual information (MI) is a measure that quantifies the degree of dependency between two variables. It indicates how much the information from one variable reduces the uncertainty of another [183]. In information theory, mutual information is used to quantify the amount of information shared between two random variables, as shown in Equation (6). Here, *P*(A, B) represents the joint probability distribution of A and B, while *P*(A) and *P*(B) are the marginal probability distributions of A and B. Let us take *I*(A, B) as an example for explanation. Since A and B are relatively independent and can be distinguished as “high” or “low” states, we can calculate probabilities like *P*(A_high_, B_high_), *P*(A_high_, B_low_), and *P*(A_low_, B_high_), *P*(A_low_, B_low_). The calculation of the marginal probability is performed as follows: *P*(A_high_) = *P*(A_high_, B_high_) + *P*(A_high_, B_low_). By calculating the joint and marginal probabilities for each pair of variables in the same way, we can use the aforementioned formula to complete the calculations.

(5) $$H\;\left(x,\;y\right)=-\sum_{i,j}p\;\left(i,j\right)\;\log\;p\;(i,j)$$

(6) $$I\;\left(X,\;Y\right)=-\sum_{x\in X}\;\sum_{y\in Y}\;P\left(x,\;y\right)\;\log\;\left(\frac{P\left(x,\;y\right)}{P\left(x\right)P\left(y\right)}\right)$$

Measuring the Shannon entropy of a concept within an organization can provide insights into the distribution and diversity of that concept in the organization [113]. For example, by quantifying the psychological entropy of individuals, we can reveal how psychological entropy is distributed across different teams or departments. This can serve as a basis for optimizing resource allocation, implementing changes, and developing plans within an organization. However, calculating Shannon entropy requires defining and quantifying the “states” or “levels” of these abstract concepts, which is a challenge in itself and offers a direction for subsequent research. Although we have proposed two methods for quantifying the psychological entropy introduced in this paper, we encourage the use of more statistical measurement methods to quantify the entropy of certain constructs in organizational psychology, thereby providing powerful quantitative tools for future empirical research.

## 7. Concluding Remarks

Since Clausius first introduced the concept of entropy in 1865, various disciplines have seen projections of the transfer and application of the entropy concept. However, in the fields of psychology and organizational management psychology, there is still no comprehensive definition and quantification method for entropy and the entropy change process. This lack hinders our in-depth study of entropy and entropy theory at the psychological level and in organizational contexts. This article adopts a positive and proactive perspective, suggesting that individuals can act as dissipative structures, proactively controlling and regulating perceived entropy, which has significant positive implications for the development of both individuals and organizations.

**Figure 2:**
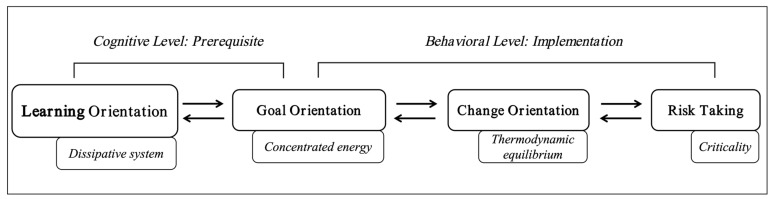
Dynamic mechanism of entropy-based proactive control model.

Specifically, we propose a four-dimensional, entropy-based proactive control model, as these four dimensions can be explained by entropy, and, simultaneously, these dimensions can explain all human organizational behaviors. In detail, first, all human activities can be understood as learning processes; humans must grow through learning, thereby achieving entropy reduction. Second, human activities must be goal-oriented; goals focus energy, maintain order, and achieve entropy reduction. Third, the realization of goals is accomplished through change, making change orientation a concrete path to achieving goals and reducing entropy. Fourth, implementing change, causing phase transitions, and fostering emergence inevitably involve facing uncertainties and encountering risks. Hence, individuals must be capable of risk taking, motivating them to pursue certain risks while avoiding others to achieve positive adaptive outcomes (as shown in Figure 2). These four dimensions collectively embody how individuals adapt to uncertain scenarios proactively, aiming to regulate and control internal entropy.

In summary, theoretically, the proposition of psychological entropy provides a new framework for understanding disorder, chaos, and uncertainty (entropy increase) within individual situations. This also offers a new theoretical method for studying dynamic changes in individuals and organizations. Moreover, the introduction of psychological entropy reflects the process of interdisciplinary integration, showcasing the potential of interdisciplinary research in explaining complex human behavior. Practically, the proposition of an entropy-based proactive control model can guide organizations in better understanding and managing employees’ behaviors and attitudes during transformational implementations, thereby motivating active participation in the organizational change process. Additionally, we believe that psychological entropy is not only a key determinant of individual potential for sustained development in organizational changes but also an important indicator for predicting various positive organizational behaviors. We believe the study and practice of psychological entropy have great potential to help engender a more orderly and harmonious world for all.

## Figures and Tables

**Table 1:** Four dimensions of an entropy-based proactive control model.

Four Characteristics of Entropy Change	Psychological Entropy
Entropy reduction occurs in open systems/dissipative systems	Learning orientation
The higher the concentration of energy, the lower the entropy	Goal orientation
The equilibrium state has the highest entropy and the most dispersed energy	Change orientation
The complexity of the critical state is the highest, and entropy is higher	Risk taking

## Data Availability

No new data were created or analyzed in this study. Data sharing is not applicable to this article.
